# Molecular characteristics of *Staphylococcus aureus* nasal carriage among health care workers at a Referral Hospital in Zabol, Iran

**DOI:** 10.11604/pamj.2019.34.196.16645

**Published:** 2019-12-12

**Authors:** Hamid Vaez, Zahra Rashki Ghalehnoo

**Affiliations:** 1 Department of Microbiology, School of Medicine, Zabol University of Medical Sciences, Zabol, Iran

**Keywords:** Hospital-acquired infection, methicillin-resistant S. aureus, nasal carriage, S. aureus

## Abstract

**Introduction:**

*Staphylococcus aureus (S. aureus)* is known as one of the most important hospital pathogens responsible for a wide range of infections. Limited data concerning the prevalence of nasal carriage of *S. aureus* and its molecular characteristics are available in Zabol province, Iran. Therefore, the aim of the present study was to determine the prevalence of nasal carriage of *S. aureus* and its molecular characteristics isolated from health care workers (HCWs).

**Methods:**

Totally, 251 nasal swabs were collected from HCWs at a referral hospital, from March to September 2017. Disk diffusion method was used to identify Methicillin-Resistant *S. aureus* (MRSA). PCR amplification method was used for the detection of following genes; *sea, seb, sec, sed, see, tst, eta, etb, lukF-PV, lukS-PV* and SCCmec types.

**Results:**

Of 251 collected swabs, 31 (12.4%) cases were identified as *S. aureus* carriers, which 14 (14/31; 45.2%) isolates were MRSA. The most prevalent detected genes were *sea* and *tst*, with 22.6% and 9.7%, respectively. The most prevalent SCCmec type was SCCmec type IV (28.6%).

**Conclusion:**

We found that the prevalence of MRSA nasal carriage is at high level and must be considered as a significant health care problem at the investigated hospital. Strict implementation of infection-control policies and rational use of antibiotics are the main pillars for controlling the spread of *S. aureus* at hospital.

## Introduction

*Staphylococcus aureus (S. aureus)* is recognized as one of the most important community-acquired and hospital-associated pathogens that is responsible for a wide range of infections including blood stream infections, soft tissue infections, pneumoniae and urinary tract infections [1]. In human, *S. aureus* colonizes various sites of the body; however, the anterior nares are the main ecological niches for *S. aureus* and it has been reported that 20-30% of individuals are persistent carriers of *S. aureus* and around 30% are transient carriers [2]. *S. aureus* can evade host immune system defense barriers by producing different enzymes and toxins. In fact, a strong correlation between toxins and disease has been reported. The main *S. aureus* toxins have been divided into following three groups; 1) pore-forming toxins (Hemolysin-α, Hemolysin-β, Leukotoxin and γ- Hemolysin), 2) exfoliative toxins (ETs) and 3) superantigens toxins (toxic shock syndrome toxin and the staphylococcal enterotoxins) [3]. When first introduced, penicillin has a substantial impact on *S. aureus*, however, it was gradually replaced with other beta-lactam antibiotics due to the emergence of beta-lactamase producing strains of *S. aureus* [4,5]. Methicillin was introduced to treat infection caused by penicillin resistant isolates, but occurrence of resistance for isolates of *S. aureus* (MRSA) to methicillin resulted in reducing the efficacy of antibiotic. In fact, MRSA isolates harbor *mecA* gene responsible for production of modified penicillin-binding-protein (PBP) called PBP2a with low affinity for beta-lactam antibiotics [4,5]. This resistance gene (*mecA*) is located on a genetic element called staphylococcal cassette chromosome (SCC). Based on genetic elements and composition, two types of MRSA have been identified, community acquired methicillin-resistant *S. aureus* (CA-MRSA) and hospital acquired methicillin-resistant *S. aureus* (HA-MRSA). Five main types of SCCmec have been characterized. Types I, II and III are the most prevalent types in HA-MRSA, whereas types IV and V are the most often associated with CA-MRSA [4,5]. Health care workers (HCWs), identified as nasal carrier of *S. aureus*, play an important role in the transmission of *S. aureus* infections within and between wards [6]. To reduce the spread of *S. aureus* infections for hospital screening of HCWs and obtain knowledge about virulence factors, genetic diversity can provide useful information for adapting infection control programs. Therefore, the aim of this study was to determine the prevalence of nasal carriage of *S. aureus* and its molecular characteristics isolated among HCWs at a referral hospital in Zabol province, southeast of Iran.

## Methods

**Study design and setting:** this cross-sectional study was carried out from March to September 2017 at Amir-al-momenin hospital affiliated to Zabol University of Medical Sciences, Iran. This hospital is the only referral hospital in the region with 224 beds, serving about four hundred thousand people.

**Sample collection and bacterial identification:** we collected samples from staffs who were consent to participate in the study. Participants were excluded if they had history of antibiotics consumption in three weeks prior to sample collections. Sterile cotton swab was used to collect sample from nasal cavity of participated HCWs. For all participants, sex and job were recorded. Nasal swabs were immediately inoculated onto Mannitol Salt Agar (Himedia, India). After 24-48 hours incubation at 37^o^C, *S. aureus* were identified by common standard microbiological tests including catalase, Gram staining, DNase and coagulase [7].

**Antimicrobial susceptibility testing:** to identify MRSA isolates, disk diffusion method (cefoxitin 30 µg, MAST, UK) was used according to Clinical Laboratory Standard Institute (CLSI) guidelines [8]. All isolates were confirmed by *mecA*-specific primers and PCR [9].

**Molecular identification of SCCmec types by multiplex-PCR:** bacterial genomic DNAs were extracted according to previously described methods [5]. All *mecA* positive isolates were subjected to SCCmec typing by using previously designed primers and conditions [10].

**Molecular detection of virulence genes:** the PCR amplifications were carried out on extracted DNAs for the detection of following genes; *sea, seb, sec, sed, see, tst, eta, etb, lukF-PV* and *lukS-PV* [11,12]. All PCR amplifications were carried out by Ampliqon (Denmark) ready to use master mix. The PCR products were separated by electrophoresis on a 1% agarose gel. Separated bands were stained with Sybr safe (Thermo Fisher Scientific Inc.) and visualized picture was captured on Gel-documentation system (Uvitec, UK).

**Statistical analysis:** data entry and statistical analysis were performed using SPSS version 16 (SPSS Inc., Chicago, IL) software and Chi-square or exact Fisher´s tests. P < 0.05 was considered statistically significant.

**Ethical considerations:** this study was approved by the Ethics committee of Zabol University of Medical Sciences, Zabol, Iran (Code: zbmu.1.REC.1396.34).

## Results

In this study, 251 nasal swabs were totally collected from HCWs. Of 251 taken swabs, 180 (71.7%) and 71 (28.3%) ones belonged to female and male, respectively. The most percentage of samples were taken from nurses with 59.3% (149), followed by midwives with 14.4% (36), operating room technicians with 12% (30), laboratory staffs with 9.6% (24), nurse assistants with 4.4% (11) and physicians with 0.3% (1) of total samples. Among these samples, 31 (12.4%) cases were *S. aureus* carriers including nurses 15 (15/149; 10%), laboratory staffs five (5/24; 20.1%), midwives four (4/36; 11.1%), operating room technicians three (3/30; 10%), nurse assistants three (3/11; 27.2%) and physicians one (1/1; 100%). Out of 31 isolated *S. aureus* 14 (14/31; 45.2%) isolates were MRSA. As shown in Table 1, the results of PCR analysis revealed that 21.4% of MRSA and 23.5% of methicillin-susceptible *S. aureus* (MSSA) were sea positive (p =0.8). The prevalence of *tst* among MRSA and MSSA isolates were 14.3% and 5.8%, respectively (p = 0.4). Other virulent genes were not detected. The results of SCCmec typing showed that 28.6% and 7.1% of MRSA harbored SCCmec type IV and I, respectively. The rest of the isolates were untypable.

**Table 1:** Molecular characteristics of MRSA and MSSA from nasal carriage of HCWs

Characteristics Isolates	*sea*	*seb*	*sec*	*sed*	*see*	*tst*	*eta, b*	*luk*
MRSA	3 (21.4%)	ND	ND	ND	ND	2 (14.3%)	ND	ND
MSSA	4 (23.5%)	ND	1 (5.9%)	ND	ND	1 (5.8%)	ND	ND
Total	7 (22.6%)		1 (3.2%)			3 (9.7%)		

## Discussion

*S. aureus* is one of the most important nosocomial pathogens and is the leading cause of life-threating infections such as bacteremia, pleuropulmonary, infective endocarditis and soft tissue infections [13]. The transmission of *S. aureus* at hospitals is usually occurred by direct skin-to-skin contact or indirect contact via contaminated medical devices and surfaces [14]. Regarding to the important role of HCWs in performing health-care practices, rapid identification of HCWs carriers will result in reduction of life-threating infections caused by this microorganism. To best our knowledge, the prevalence of *S. aureus* nasal carriage among HCWs in Zabol province has not been determined and this is the first report. In this study, nasal carriage of *S. aureus* was identified in 12.4% of studied HCWs. This prevalence is lower than previous reports from different provinces of Iran. For example, a study conducted by Askarian *et al*. in Shiraz province, southwest of Iran, showed that 31% of studied HCWs were *S. aureus* carriers [15]. Furthermore, in Isfahan and Ilam provinces, the prevalence of *S. aureus* among HCWs was 19% and 37%, respectively [4,16]. A comprehensive study conducted by Emaneini *et al.* revealed that the average mean prevalence of HCWs carriers in different provinces of Iran was 22.7% [2]. It has been reported that differences in nasal carriage rate of *S. aureus* are dependent on different factors, such as age, alcoholism, chronic disease, as well as variations in sample size, identification methods and infection control measures [2,4]. Based on our results, the prevalence of MRSA was 45.2%. This finding is remarkably higher in comparison with study conducted by Khanal *et al*. (21.9%) and Dulon *et al*. (0.2% -14.5%) in Nepal and European countries, respectively [17,18]. Moreover, similar studies conducted in other provinces of Iran revealed that the prevalence of MRSA was between 30% and 77% [2,19,20]. There are several factors that may explain these differences, for example, sample size, culture techniques, infection control measures, such as hand hygiene, limited infrastructure, lack of sufficient personnel protective equipment and most important of all, lack of sufficient knowledge about transmission routes are known to be the most important reasons [21]. In this study the prevalence of the most important virulence factors of *S. aureus* was investigated. Our results revealed that the most prevalent virulence factor was enterotoxin A (22.6%) followed by toxic shock toxin (9.7%) and enterotoxin C (3.2%). Other virulence genes such as *seb, sed, see, eta* and *etb* were not detected. These results are in agreement with other reports from Malaya and Spain [22,23]. We did not identify any significant correlation between the existence of virulence factors and resistance to methicillin. However, other studies have suggested significant association between enterotoxin and resistance to methicillin, and have reported that *etb* was more prevalent in MRSA and eta was mainly prevalent in MSSA [24]. This discrepancy can be related to origin of bacteria, selective pressure and host conditions [23,25,26]. For example, results of other study conducted by Schlievert *et al*. revealed that some clonal types of *S. aureus* with as specific genotypes are mostly prevalent in severe infections and are contributed to poor patients' outcomes [25].

## Conclusion

We found that the prevalence of *S. aureus* nasal carriage at the investigated hospital is lower than most provinces of Iran. However, the prevalence of MRSA is alarmingly high and must be taken into consideration for designing infection control programs. This high rate nasal MRSA carriage among HCWs of investigated hospital can be attributed to misuse of antibiotics, poor compliance to hand hygiene, and ineffective implementation of infection control rules. Our results revealed that in order to prevent infection by *S. aureus* with considerable virulence factors at the investigated hospital constant monitoring and appropriate eradication of nasal carriage must be performed.

### What is known about this topic

- *Staphylococcus aureus* strains are considered a serious public health concern worldwide, causing different kind of infections including blood stream infections, wound infections and pneumoniae;
- Methicillin-resistant *Staphylococcus aureus* (MRSA) is identified as a hospital-acquired pathogen that due to production of different virulence factors and emergence of several antibiotic resistance mechanisms are associated with high mortality and morbidity in hospitals.

### What this study adds

- The prevalence of methicillin-resistant *Staphylococcus aureus* strains in Zabol hospital is alarmingly high;
- Molecular analysis revealed that sea and tst were the most prevalent virulence factors in identified *S. aureus* strains;
- Staffs of investigated hospital could be potential sources of MRSA, therefore elimination of strains from nasal carriage can hamper spread of MRSA infections in Zabol hospital.

## Competing interests

The authors declare no competing interests.
